# Evolving Stark Effect During Growth of Perovskite Nanocrystals Measured Using Transient Absorption

**DOI:** 10.3389/fchem.2020.585853

**Published:** 2020-10-15

**Authors:** James C. Sadighian, Kelly S. Wilson, Michael L. Crawford, Cathy Y. Wong

**Affiliations:** ^1^Department of Chemistry and Biochemistry, University of Oregon, Eugene, OR, United States; ^2^Oregon Center for Optical, Molecular, and Quantum Science, University of Oregon, Eugene, OR, United States; ^3^Materials Science Institute, University of Oregon, Eugene, OR, United States

**Keywords:** perovskite, nanocrystals, surface, ultrafast, transient absorption, spectroscopy, Stark effect

## Abstract

Methylammonium lead triiodide (MAPbI_3_) nanocrystals (NCs) are emerging materials for a range of optoelectronic applications. Photophysical characterization is typically limited to structurally stable NCs owing to the long timescales required for many spectroscopies, preventing the accurate measurement of NCs during growth. This is a particular challenge for non-linear spectroscopies such as transient absorption. Here we report on the use of a novel single-shot transient absorption (SSTA) spectrometer to study MAPbI_3_ NCs as they grow. Comparing the transient spectra to derivatives of the linear absorbance reveals that photogenerated charge carriers become localized at surface trap states during NC growth, inducing a TA lineshape characteristic of the Stark effect. Observation of this Stark signal shows that the contribution of trapped carriers to the TA signal declines as growth continues, supporting a growth mechanism with increased surface ligation toward the end of NC growth. This work opens the door to the application of time-resolved spectroscopies to NCs *in situ*, during their synthesis, to provide greater insight into their growth mechanisms and the evolution of their photophysical properties.

## 1. Introduction

Hybrid organic-inorganic perovskite nanocrystals (NCs) are currently the focus of significant interest owing to their potential applications in optoelectronic devices. Their large absorption coefficients (Fu et al., 2015), high defect tolerance (Dirin et al., 2016), excellent photoluminescence quantum yield (PLQY) (Hassan et al., 2019), and potential for low-cost, facile production (Protesescu et al., 2015) coupled with a narrow, tuneable emission spectrum (Hassan et al., 2016) has driven a boom of research in the synthesis and characterization of these materials. These NCs are ordinarily grown through either a hot injection or reprecipitation style synthesis. In these solution-based syntheses the reaction is initiated when dissolved precursor reaches a critical threshold to cause LaMer nucleation (LaMer and Dinegar, 1950). Following this, NCs are allowed to grow until the desired size and morphology is reached. The morphology (Pan et al., 2016; Sun et al., 2016), stability (Huang et al., 2017), and photophysics (Peterson et al., 2014; Teunis et al., 2017) of NCs are strongly dependent on the surface owing to their large surface-to-volume ratios. Surface atoms lacking bonds to capping ligands exhibit localized electronic states with energies that can lie within the band gap. These mid-gap states act as traps for excited electrons or holes, suppressing radiative recombination and hampering performance in light emitting devices (Boles et al., 2016).

**Figure 1:**
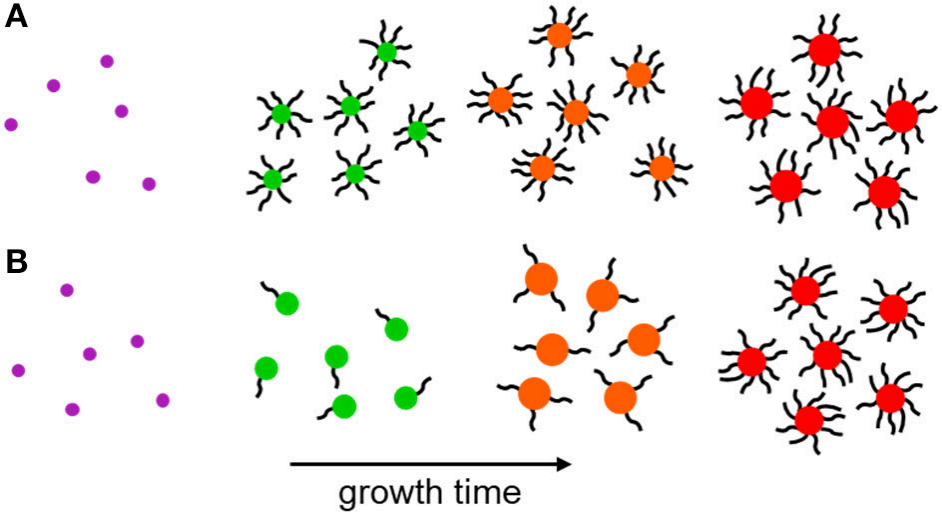
Schematic of NC growth. **(A)** Following LaMer nucleation the immature NCs are well-capped by surface ligands throughout most of their subsequent growth. **(B)** Nascent NCs nucleate following the same LaMer-type mechanism, but surface ligation occurs primarily after NC growth. A terminal ligation stage is proposed in literature for CdSe and MAPbI_3_ NCs and is supported by the measurements in this work.

The quality of the NC surface during growth is still poorly understood and the timescales of nucleation and growth are prohibitively short for investigation using typical surface characterization techniques, such as X-ray photoelectron spectroscopy (Katari et al., 1994), electron energy loss spectroscopy (Wang et al., 1998), small-angle X-ray scattering (Mattoussi et al., 1998), and 2D nuclear magnetic resonance techniques (De Roo et al., 2016). Recently, use of a solvation-mediated synthesis (Wang et al., 2017), coupled to a rapid sampling technique (Sadighian et al., 2019), permitted the measurement of linear absorbance and fluorescence during growth. This study revealed that NCs initially grow in size while their surfaces remain poorly-capped by passivating ligands, and do not become well-capped until they are almost fully grown (Figure 1) (Sadighian et al., 2020). Visible absorbance and fluorescence measurements report on transitions from the ground and emissive band-edge states, respectively. The peak positions and lineshapes can provide insight into the NC size distribution, and fluorescence intensity is often used to infer the degree of NC surface passivation. However, these spectroscopies are insensitive to other important transitions, such as carrier trapping and non-radiative recombination, and the dynamics of the excited carriers. A comprehensive understanding of how NC photophysics evolves during synthesis may provide deeper insights into NC growth mechanisms, the nature of the NC surface, and how a synthesis can be tuned to achieve desired morphologies and optoelectronic properties.

In this paper, we demonstrate a technique that can provide further insight into the evolving NC surface by probing the electric field generated by carriers localized at surface traps. Photogenerated electron-hole pairs become spatially separated when a carrier is trapped at these surface sites, creating an electric field inside the NC. The presence of an electric field can modulate the optical transitions of an NC *via* the Stark effect (Colvin and Alivisatos, 1992; Colvin et al., 1994; Klimov, 2000; Sharma et al., 2019). Analysis of the modulated absorbance spectrum lineshape can provide insight into the electric fields in the NCs (Bublitz and Boxer, 1997). The quantum-confined Stark effect (QCSE) changes the bandgap transition energy by shifting the electron and hole energy levels (Walters et al., 2018). This typically redshifts the bandedge absorption and causes the differential absorbance spectrum to exhibit the lineshape of the first derivative of the linear absorbance. In systems that lack any specific orientation, such as randomly distributed surface traps on NCs, the internal electric field generated by spatially separated, trapped carriers results in a population of randomly oriented dipoles in the sample. This would act to inhomogenously broaden the overall transition, and as a result the differential absorbance spectrum would resemble the second derivative of the linear absorbance (Tanaka and Kondo, 2003; Queloz et al., 2020).

Electroabsorbance measurements of 2D hybrid perovskites have exhibited lineshapes that could be fit to a weighted sum of first and second derivatives of the absorbance spectrum (Queloz et al., 2020). These two components were assigned to a spectral redshift arising from a QCSE and broadening due to loosely-bound, screened electron-hole pairs, respectively. This same lineshape was observed upon photogeneration of charge carriers in these perovskites during transient absorption (TA) measurements. This indicates that the presence of spatially separated electrons and holes in surface traps can cause internal electric fields that yield lineshapes characteristic of the Stark effect. Thus, the Stark lineshape measured by TA can report on the surface quality of NCs.

TA is a powerful time-resolved spectroscopy that has been used to understand excited state processes such as Auger recombination (Klimov and McBranch, 1998; Guyot-Sionnest et al., 1999), energy transfer to phonons (Urayama et al., 2001) or ligands (Guyot-Sionnest et al., 2005; Li et al., 2019), and carrier trapping (Mondal and Samanta, 2017) in NCs. Typically, this pump-probe technique is performed by varying the path length of one pulse relative to the other by use of a retroreflector on a motorized translation stage. The transmission of many successive laser pulses is recorded at each pump-probe time delay in series. This technique typically requires measurement timescales on the order of tens of minutes to several hours, depending on factors such as sample response and laser noise. As a result, in its typical implementations TA fails to accurately report on excited state dynamics in non-equilibrium systems that are chemically changing on timescales shorter than a few hours, such as growing NCs.

**Figure 2:**
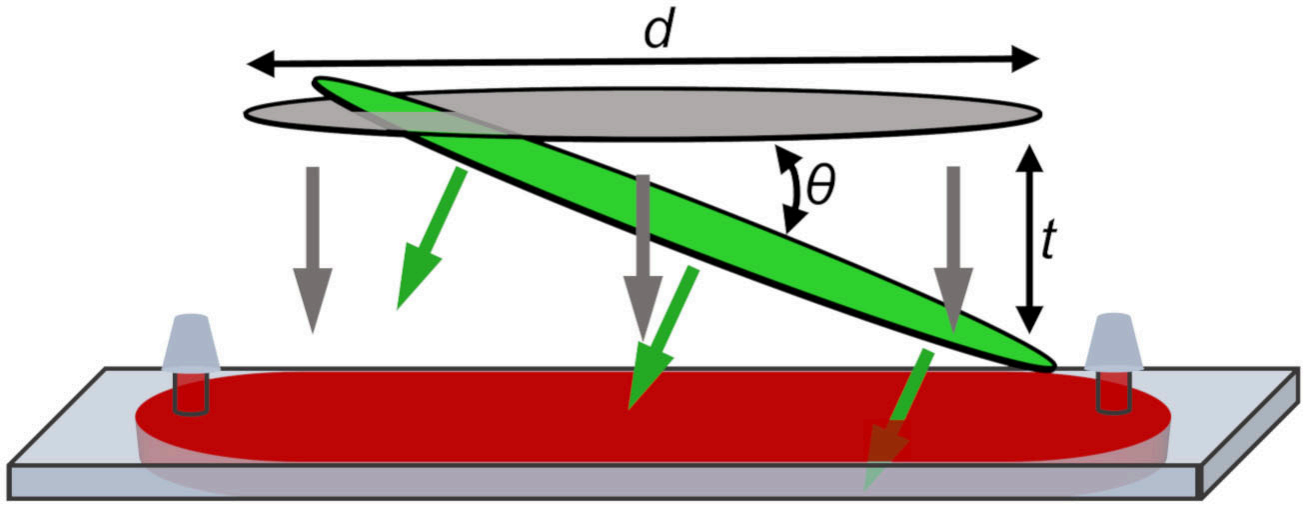
Sample plane in SSTA instrument showing pump (green) and probe (gray) focused to lines. Probe pulse is incident normal to the cuvette. Spatially encoded time delay, *t*, is generated by the angle of the pump pulse relative to the probe pulse, θ, and the length of overlap between the two pulses, *d*. Sample is injected into the flow-cell cuvette through septa to prevent solvent evaporation during the measurement.

TA measurements can be conducted more rapidly by using a single-shot transient absorption (SSTA) spectrometer that enables an entire transient to be recorded from a single pump-probe pulse pair. This can be achieved by tilting the wavefront of the pump pulse relative to the probe (Fourkas et al., 1995; Makishima et al., 2006). In this case, the time delay range is determined by Equation (1):

(1) $$\begin{array}{r} t_{range}=\frac{dsin\left(\theta \right)}{c} \end{array}$$

where *d* is the length of overlap between pump and probe pulses, θ is the angle between the tilted pump pulse and the probe pulse, and *c* is the speed of light (Figure 2). Here, we use a recently developed broadband SSTA spectrometer (Wilson and Wong, 2018; Wilson et al., 2020) to track the evolution of exciton dynamics in methylammonium lead triiodide (MAPbI_3_) perovskite NCs as they nucleate and grow and as their surfaces are passivated with ligands. A complete TA spectrum with excellent signal to noise can be collected using this instrument in less than 1 min, allowing us to accurately measure immature NCs before they degrade (Sadighian et al., 2019). As a result, we are able to observe the surface of NCs being capped in real time by monitoring the evolving Stark lineshape. A carrier that has been photoexcited by the pump may localize on a surface trap state, creating an electric field within the NC. Using differential measurement, the probe then reports the effect of an ensemble of these electric fields on the absorption of the NC sample. These findings agree with previous reports of the growth mechanism of CdSe (Teunis et al., 2017) and MAPbI_3_ (Sadighian et al., 2020) NCs, and open up a new avenue for studying the surface of these materials during growth.

## 2. Methods

### 2.1. Materials

All reagents were used as received: lead iodide (99.999%, trace metals basis, Sigma-Aldrich), methylammonium iodide (MAI, ≥99%, anhydrous, Sigma-Aldrich), octylamine (99%, Sigma-Aldrich), oleic acid (90%, technical grade, Sigma-Aldrich), and hexane (≥95%, laboratory reagent grade, Sigma-Aldrich). Cresyl violet (62%, J.T. Baker) in methanol (99.8%, Certified ACS, Fisher) was used to calibrate the beam profile and spatially encoded time delay of the SSTA spectrometer.

### 2.2. Nanocrystal Synthesis

MAPbI_3_ NCs were synthesized using a previously reported solvation-limited synthesis (Sadighian et al., 2019, 2020). 460 mg of PbI_2_ and 127 mg of MAI were combined with 40 mL of hexane in a glass test tube and suspended in an ultrasonication bath (VWR, 97043-992) to provide constant mixing. The reaction was initiated with the simultaneous introduction of 150 μL octylamine and 300 μL oleic acid, and the recorded reaction time is in reference to this addition. These organic ligands act to solubilize PbI_2_ and MAI, which are otherwise insoluble in hexane (Wang et al., 2017). A recirculating chiller (VWR, 1165) in a closed-loop configuration with an aluminum block was used to maintain a temperature of 22°C in the ultrasonication bath. An HDPE syringe was used to withdraw aliquots of the reaction mixture at selected time points. Each aliquot was filtered through a syringe filter (VWR) with a 0.45 μm pore polytetrafluoroethylene (PTFE) membrane and into a 0.2 mm path length quartz flow cell cuvette (Starna Cells, 48-Q-0.2). Following the 15 min mark a 1.0 μm PTFE pre-filter (Whatman Rezist) was used in conjunction with the 0.45 μm filter to compensate for increased suspended particulate. An additional 5.0 μm filter (Whatman Rezist) was added after 60 min. The flow cell was emptied, rinsed with acetone and hexane, and dried with a stream of nitrogen before each successive measurement.

### 2.3. Absorbance and Fluoresence

Absorbance and fluorescence of the filtered NC aliquots were simultaneously recorded on a homebuilt spectrometer (Supplementary Figure 1) using the same cuvette and sample described above. To measure absorbance, light from a tungsten-halogen lamp (Thorlabs, SLS201) was directed into the sample using a fiber optic cable (Thorlabs, M28L01) and the resulting transmission collected using a second fiber. A 405 nm laser (Thorlabs, CPS405) was used as the fluorescence excitation source. Emitted light was collected using a fiber optic cable (Thorlabs, M95L01) directed to the spot upon which the laser was incident on the cuvette and angled to avoid the specular reflection of the excitation source. Absorbance and fluorescence spectra were recorded using an Ocean Optics Flame-T-VIS-NIR and Flame-T-UV-VIS spectrometer, respectively. The spectrometers were operated using a homebuilt Python software package. Absorbance and fluorescence were recorded immediately before and after collecting SSTA measurements of each aliquot to make sure the spectra did not change significantly during the measurement. The pairs of spectra were then averaged together for analysis.

### 2.4. Single-Shot Transient Absorption

SSTA measurements were performed using a previously described homebuilt instrument (Wilson and Wong, 2018; Wilson et al., 2020). A 1 kHz Ti:sapphire laser (Coherent, Astrella) with an 800 nm output was used to pump an optical parametric amplifier (Light Conversion, Topas Prime Plus) to generate 520 nm pump pulses that were compressed to 50 fs using a prism pair. A 2 m focal length mirror focused part of the 800 nm fundamental in a 1.6 m homebuilt gas cell with 1.5 mm quartz windows and containing 0.55 bar differential pressure of argon (PurityPlus, 99.999%) to generate broadband probe pulses. The spectral profiles of both pulses are shown in Supplementary Figure 2. The pump and probe pulses were optically chopped at 250 and 125 Hz, respectively. The addition of a chopper in the probe line enabled the subtraction of background signals arising from pump induced fluorescence, scatter, stray light, and dark current from the camera (Wilson et al., 2019). A spatial light modulator (Meadowlark, 1920 × 1152 XY Phase Series SLM) placed after the choppers was used to reshape both beams to a flat-top intensity profile to provide a uniform excitation density across the entire spatially encoded time delay range.

The pump pulse energy at the sample was set to 410 nJ to prevent non-linear interactions. The pump and probe beams were focused to lines using cylindrical lenses with focal lengths of 200 mm and 150 mm, respectively, and overlapped on a 20 μm × 22 mm area of the cuvette. While the probe was incident normal to the sample, the pump beam was tilted at an angle of 55.5° to achieve a spatially encoded time delay of 60 ps. The probe beam at the sample plane was imaged onto the slit of a grating spectrograph (Princeton Instruments, Isoplane 160), where it was measured to be 10 nJ. The probe beam was slightly defocused at the sample plane such that the entire measured wavelength range overlaps well onto the slit of the spectrograph with sufficient intensity. The spectrograph was coupled to a CMOS camera (Andor, Zyla 5.5) with a 1.3 ms exposure time which acquires 180 x 2560 pixel (1.17 × 16.6 mm) images, with the signal at each pixel corresponding to a pump-probe time delay of 24 fs. One axis of the pixel array recorded wavelength resolution of the probe and the other captured the spatially encoded time delay. Each SSTA spectrum was recorded for 60 s to maximize signal-to-noise ratio while still avoiding sample degradation. The SSTA spectrometer was operated using homebuilt Python software. Spectral calibration was performed using a HgAr calibration source, which accounts for spherical aberrations in the imaging setup through the spectrometer. Calibration of the spatially encoded pump-probe time delay was performed using SSTA measurements of cresyl violet in methanol in the same cuvette used for the NC measurements. This process corrects for chirp in the broadband probe pulse. Both the wavelength and time delay calibrations are discussed in detail elsewhere (Wilson et al., 2020).

## 3. Results and Discussion

**Figure 3:**
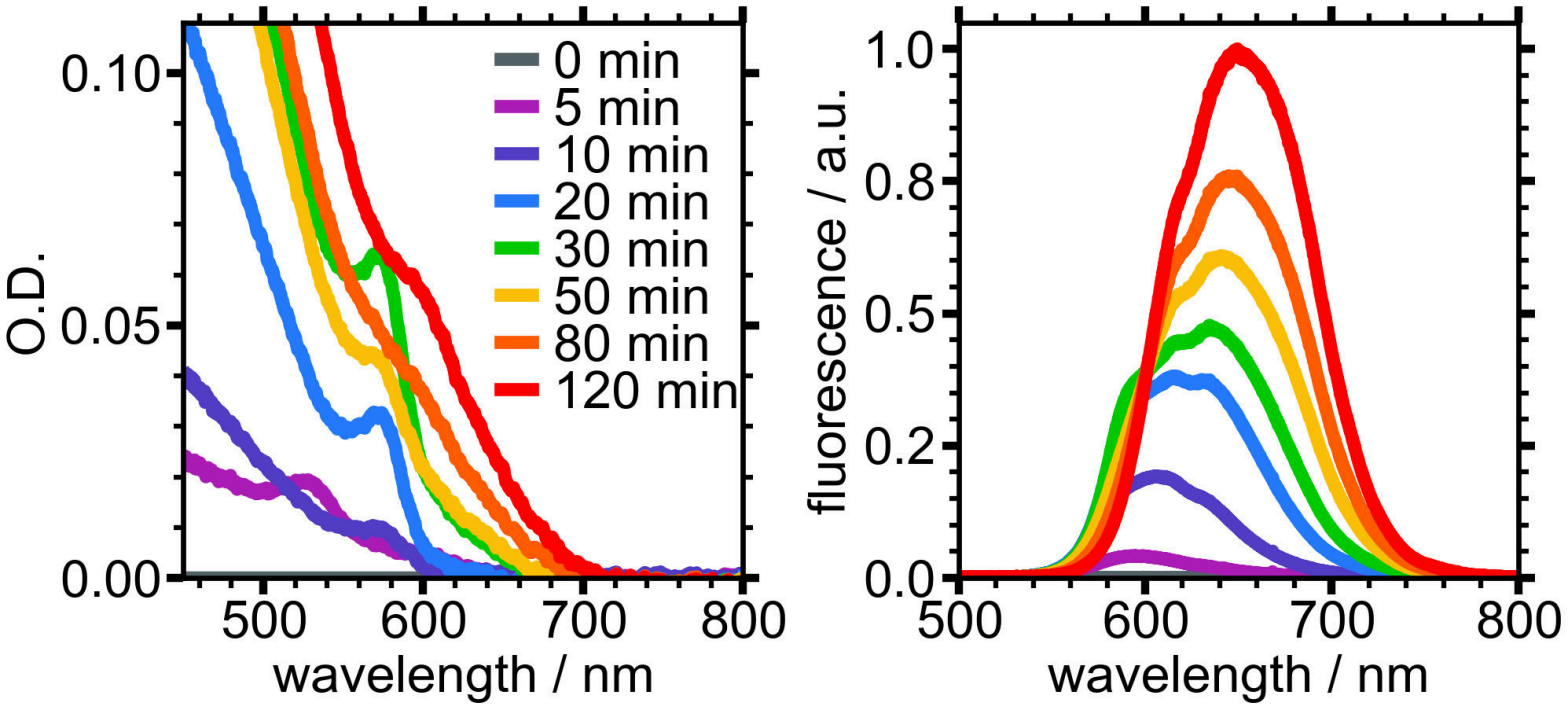
Absorbance **(Left)** and fluorescence **(Right)** of PTFE-filtered reaction mixture sampled at different times during growth.

Absorbance and fluorescence spectra at various reaction timepoints show the evolving physical and electronic structure of PTFE-filtered NCs over 120 min of growth (Figure 3). Nucleation occurred within the first 5 min, evidenced by the appearance of a broad, low intensity emission centered around 595 nm and weak absorbance near 525 nm. The fluorescence of the reaction mixture significantly increased in intensity by the 10 min mark and began to exhibit two distinct peaks. A feature emerged at 575 nm in the absorbance spectrum, which we ascribe to nascent NCs. These absorbance and fluorescence features continued to grow in intensity, reaching a maximum at the 30 min mark. Following this, the sharp absorbance peak at 575 nm began to disappear and gave rise to a broad shoulder at 610 nm, indicative of the small, nascent NCs growing larger. Likewise, the fluorescence spectrum began to lose intensity at shorter wavelengths while the peak at 635 nm continued to grow and redshift until the final measured timepoint. The evolution of these spectra are in agreement with previously reported experiments performed under similar conditions (Sadighian et al., 2019, 2020).

**Figure 4:**
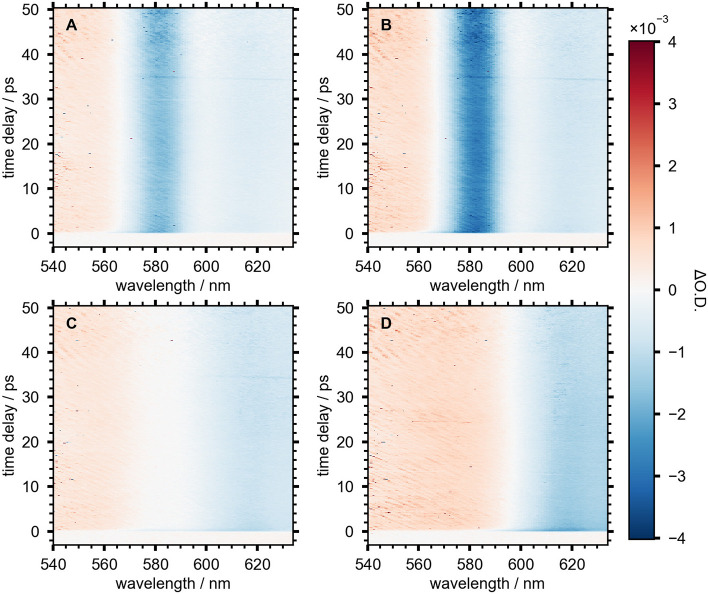
SSTA spectra of NC aliquots measured after **(A)** 20 min, **(B)** 30 min, **(C)** 50 min, and **(D)** 120 min after starting the reaction.

Select SSTA spectra for NCs at various stages of growth are shown in Figure 4. For each sample, the transient spectrum redshifts approximately 10 nm during the first 500 fs as a result of carrier cooling (Righetto et al., 2020). The spectra are quite stable for the remainder of the measured 60 ps time window. The spectrum of NCs grown for 120 min (Figure 4D) is typical of MAPbI_3_ perovskite NCs (Wang et al., 2017). The negative TA at wavelengths longer than 600 nm overlaps with the band-edge absorbance and the emission spectrum. This feature is typically ascribed to a combination of stimulated emission (SE) and ground-state bleach (GSB). The signal at shorter wavelengths is broad and positive, indicating a photoinduced absorption (PIA) to higher electronic states. The SSTA spectra of NCs grown for 20, 30, and 50 min (Figures 4A–C) show two distinct features not present in the 120 min spectrum; a strong, narrow, negative TA signal centered at 582 nm and a region of low signal intensity near 600 nm. This signal reached its maximum in the 30 min sample and had all but disappeared 50 min into the reaction. The negative signal at 582 nm does not coincide with the absorbance peak (575 nm) and the fluorescence spectrum has a shoulder at 595 nm, suggesting neither GSB nor SE can explain this signal.

**Figure 5:**
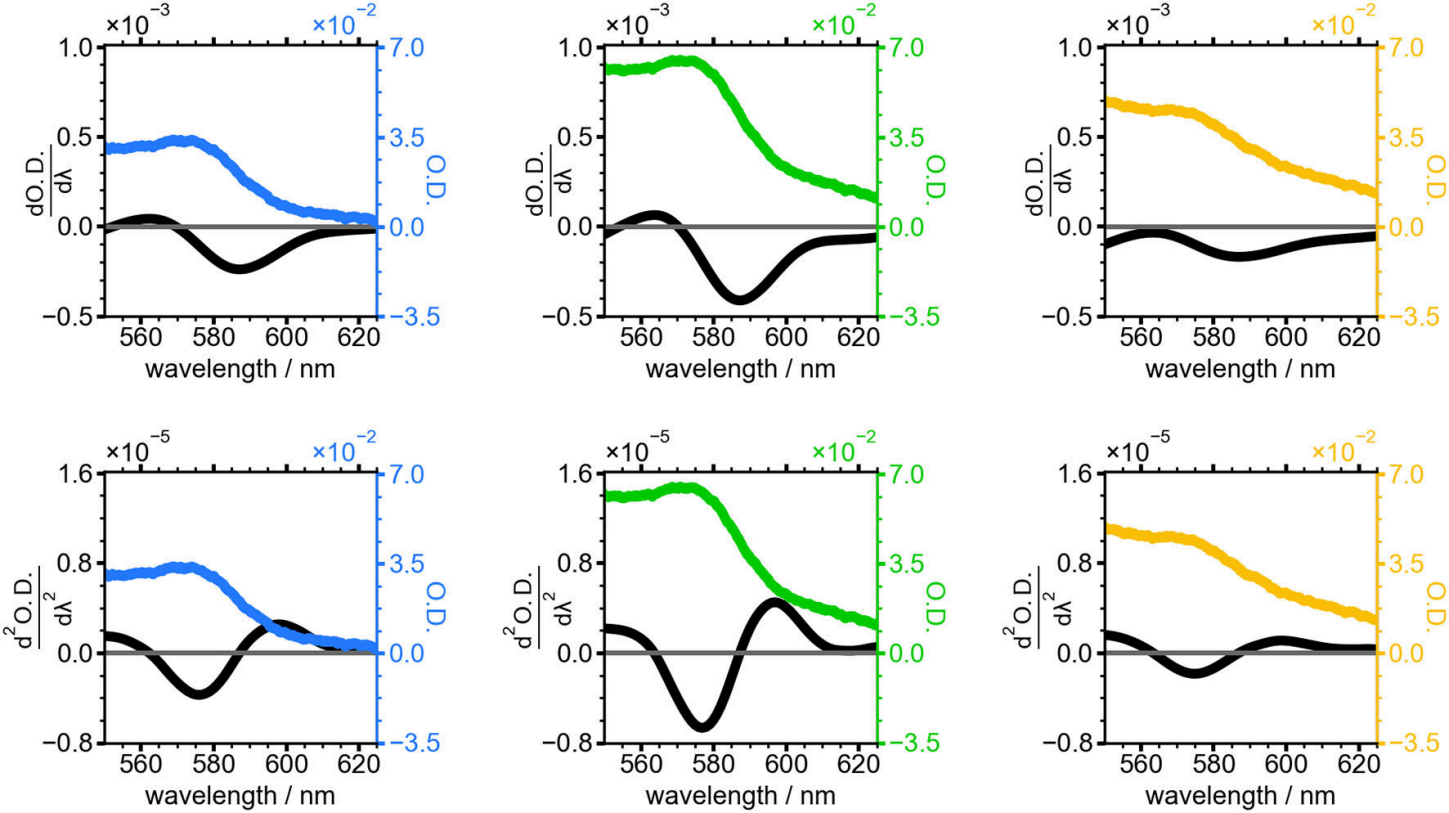
Absorbance spectra of NCs grown for 20 min (blue), 30 min (green), and 50 min (yellow). First (top) and second (bottom) derivative of the absorbance spectrum for each time point is shown in black.

First and second derivatives of the absorbance spectra for the 20, 30, and 50 min NC samples are shown in Figure 5. The lineshape of the derivatives is similar across the three selected timepoints, with the magnitude of the derivative traces reaching their maximum in the 30 min sample when the sharp absorbance peak at 575 nm is most intense. This peak is less intense and broader in width in the 50 min sample, resulting in smaller derivatives for this timepoint.

In order to elucidate the origin of the TA lineshapes and gain additional insight into the electronic structure of growing NCs, a slice of the TA spectrum, reported in differential optical density (ΔO.D.) and averaged between 5 ps and 10 ps for each growth time, *t*, was fit using Equation (2).

(2) $$\begin{array}{l} \Delta \text{O}.\text{D}.(t,\lambda )=A\frac{d\text{O}.\text{D}.(t,\lambda )}{d\lambda }+B\frac{d^{2}\text{O}.\text{D}.(t,\lambda )}{d\lambda^{2}}\\ \;+C\Delta \text{O}.\text{D}.(120\text{ min},\lambda ) \end{array}$$

**Figure 6:**
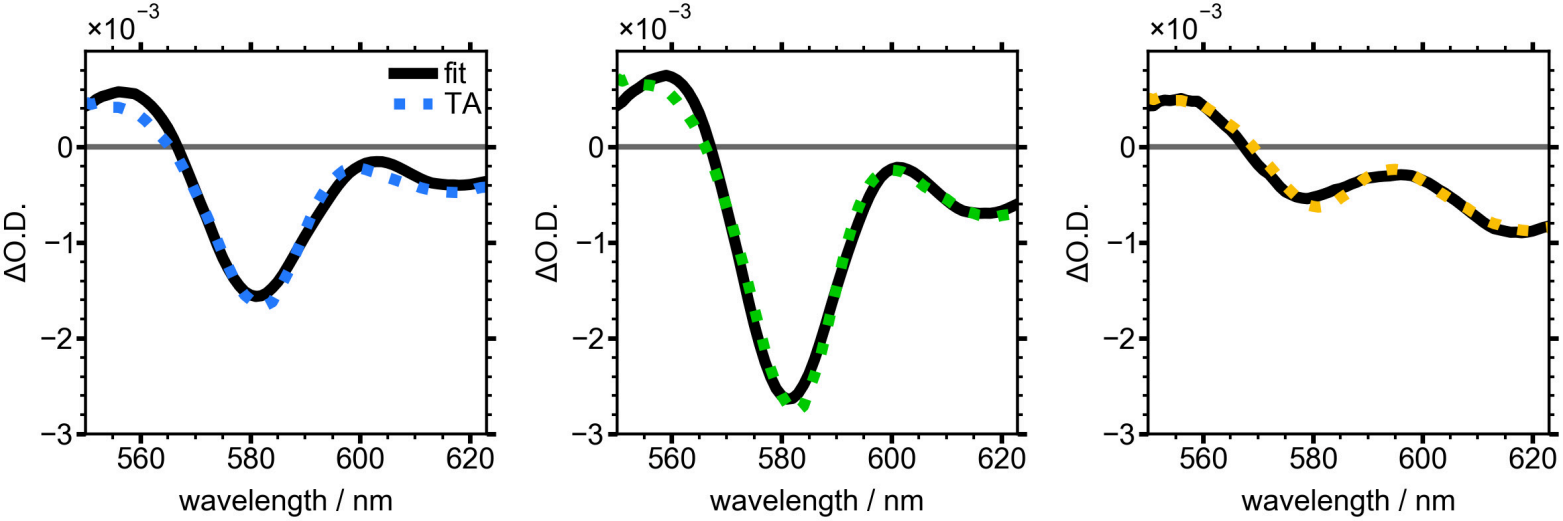
Averaged TA spectra from 5 to 10 ps for NCs grown for 20 min (blue), 30 min (green), and 50 min (yellow). Black line shows fit to Equation (2).

The first two terms represent the first and second derivatives of the absorbance spectrum at the selected growth time and the third term is the analogous TA spectrum of the NC sample after 120 min of growth. This term accounts for the contribution of well-passivated NCs to the overall TA spectrum at each timepoint. The resulting fits are overlaid with TA slices in Figure 6. The colored, dashed lines are TA slices for the three time points from Figures 4, 5, and the fits (solid black lines) show good agreement. These slices reveal the evolution of the electric field induced by electron-hole pairs generated by the pump pulse in the nascent NCs. The negative TA signal at 580 nm was clearly visible after 20 min of NC growth and reached a maximum after 30 min, indicating the presence of growing, poorly-capped NCs. During the remainder of the reaction this feature lost intensity and by 50 min was barely discernible.

**Figure 7:**
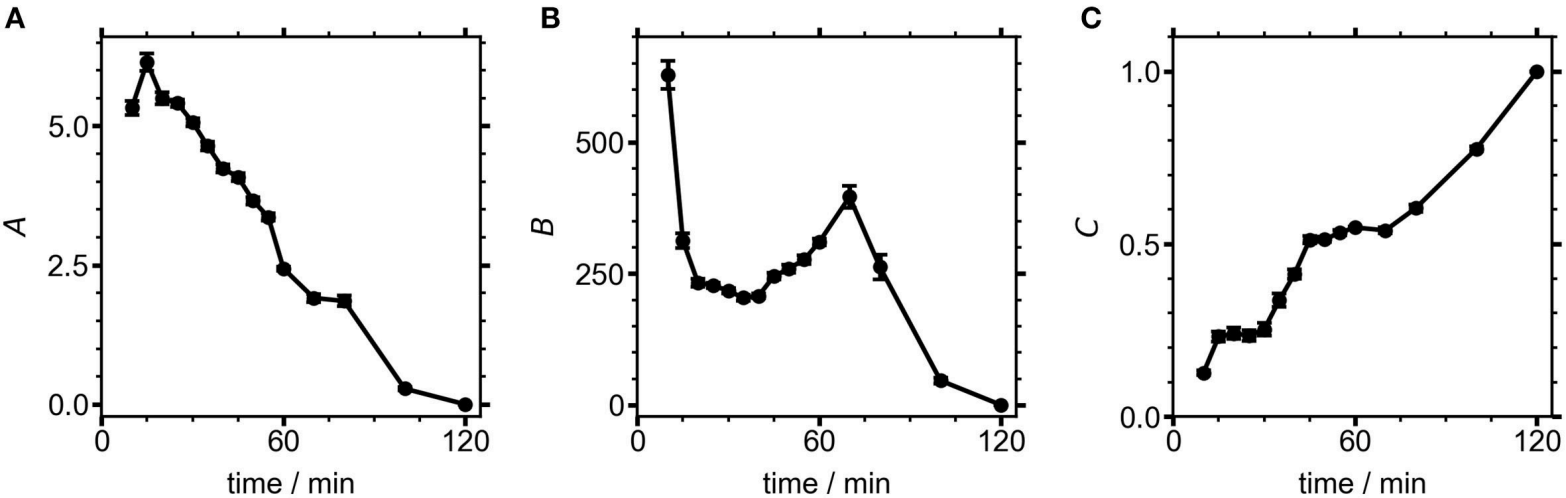
Fit coefficients from Equation (2) for NCs measured at different growth times. Contributions from the **(A)** first and **(B)** second derivatives, as well as the **(C)** 120 min NC component to the overall fit.

The values of the three coefficients from Equation (2) are displayed with fit errors in Table 1. Tracking their values during the reaction quantifies the evolving contributions to the TA lineshapes (Figure 7). The first term, *A*, relates the observed signal to the first derivative of the absorbance, which occurs when the field causes a shift in the transition energy for the NCs. Here, the presence of spatially separated electrons and holes at surface traps would induce a dipole that could stabilize the excited electronic states, potentially redshifting the optical transition. The second derivative term, *B*, has the largest contribution to the signal throughout nearly the entire measured range. This term arises from an overall broadening of the absorbance spectrum, suggesting the presence of many randomly oriented dipoles in the sample arising from surface-trapped carriers.

**Table 1:** Best-fit values for parameters *A*, *B*, and *C* with one standard deviation error of the fitted variables (σ_*X*_).

**Reaction timepoint**	***A*±σ_*A*_**	***B*±σ_*B*_**	***C*±σ_*C*_**
**(min)**			
10	5.33 ± 0.12	628 ± 27	0.127 ± 0.007
15	6.15 ± 0.15	313 ± 14	0.232 ± 0.015
20	5.49 ± 0.11	232 ± 8	0.241 ± 0.017
25	5.41 ± 0.07	227 ± 5	0.235 ± 0.015
30	5.07 ± 0.07	217 ± 5	0.253 ± 0.018
35	4.64 ± 0.08	204 ± 5	0.337 ± 0.019
40	4.24 ± 0.07	208 ± 5	0.413 ± 0.013
45	4.08 ± 0.07	245 ± 6	0.513 ± 0.011
50	3.66 ± 0.07	259 ± 8	0.514 ± 0.009
55	3.36 ± 0.06	277 ± 8	0.533 ± 0.008
60	2.44 ± 0.04	310 ± 6	0.548 ± 0.004
70	1.91 ± 0.07	396 ± 21	0.539 ± 0.009
80	1.86 ± 0.09	262 ± 23	0.604 ± 0.011
100	0.281 ± 0.03	46.6 ± 5.6	0.776 ± 0.006
120	0	0	1

The electric field produced by a trapped carrier should become smaller as a NC grows larger, so the decreasing contribution of the derivative lineshapes during NC growth could be the result of both increasing NC size and improved surface capping, resulting in fewer NCs with internal electric fields. While the contributions from both derivatives decline to zero over the course of the reaction, *B* shows a brief period of growth between 30 and 70 min into the reaction. The electric field strength at any particular time point during NC growth could be estimated from these results if the NC size were known, assuming that one carrier is surface-trapped while the other is delocalized (i.e., on average centrally located within the NC). Future work will focus on concurrent measurements of NC size during the reaction, which will enable the magnitude of the electric field caused by a surface-trapped carrier to be modeled during NC growth. This will aid in the interpretation of the rise in *B* while *A* continually decreases. *C*, the contribution of well-passivated NCs, shows a fairly linear growth throughout the entire synthesis. By the end of the reaction the NCs are well-capped with ligands, and surface traps no longer contribute to the TA signal. Thus, our measurements indicate that poorly-capped NCs are dominant during the growth of perovskite NCs, becoming progressively better capped as the growth process continues, similar to the case shown in Figure 1B. Future studies using different polarities of filter media to separate well- and poorly-capped NCs (Sadighian et al., 2020) will seek to test this assumption and further isolate the evolving lineshapes of these sub-populations within the reaction mixture. As demonstrated here by the intriguing trends in the weights of the two derivative features, the ability of SSTA to measure lineshapes during a NC synthesis provides a new avenue to deeper insights into how NCs grow. Further analyses of both the lineshapes and the exciton dynamics hold promise for understanding the evolving nature of carrier traps in nascent NCs.

## 4. Conclusion

A novel, broadband, tilted-pulse SSTA spectrometer with a 60 ps time delay was used to investigate evolving excited state dynamics in NCs grown *via* a solvation-limited synthesis. Growing NCs were found to exhibit a unique TA lineshape indicative of the Stark effect. Fits of these data to a weighted sum of linear absorbance spectrum derivatives show that this lineshape is likely caused by spatially seperated charge carriers in surface trap states. This adds to the growing body of evidence that these NCs are poorly capped during most of their growth (Teunis et al., 2017; Sadighian et al., 2020). This work proves the applicability of this technique to the study of non-equilibrium systems such as growing NCs that were previously inaccessible with non-linear spectroscopies. The development of SSTA and this sampling technique provide powerful tools for understanding how the electronic structure and excited state dynamics of NCs change during their synthesis. These types of experiments may offer new insight into NC growth mechanisms and how reaction parameters can be changed to target desired photophysics.

## Data Availability Statement

The raw data supporting the conclusions of this article will be made available by the authors, without undue reservation.

## Author Contributions

JS optimized and executed the synthesis. KW optimized and operated the instrument. JS, KW, and MC executed the experiments. JS analyzed the data. JS and CW designed the research. All authors contributed to manuscript revision.

## Conflict of Interest

The authors declare that the research was conducted in the absence of any commercial or financial relationships that could be construed as a potential conflict of interest.
