# Supplementary material for: Evolving Stark Effect During Growth of Perovskite Nanocrystals Measured Using Transient Absorption
Source: Front Chem. 2020 Oct 15;8:585853. doi: 10.3389/fchem.2020.585853 (PMC7594514; doi:10.3389/fchem.2020.585853)
Supplement: Supplementary file 1 [file Data_Sheet_1.PDF]

Supplementary Material

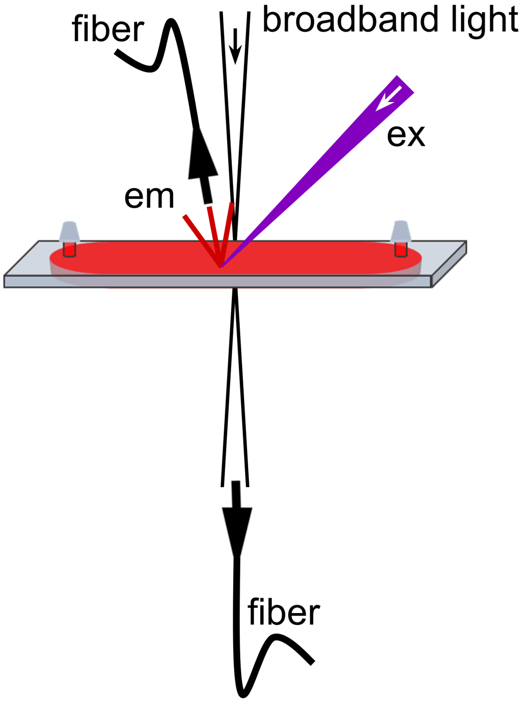

Figure S1. Schematic showing arrangement of light sources and collection fibers for absorbance and fluorescence measurements. Broadband light from a tungsten-halogen lamp (Thorlabs, SLS201) was focused into the sample cuvette and the transmission collected using a fiber optic cable (Thorlabs, M28L01). A 405 nm laser (Thorlabs, CPS405) was used as the fluorescence excitation source (ex). Emitted light (em) is collected with a fiber optic cable (Thorlabs, M95L01) angled to avoid scatter from the excitation source.

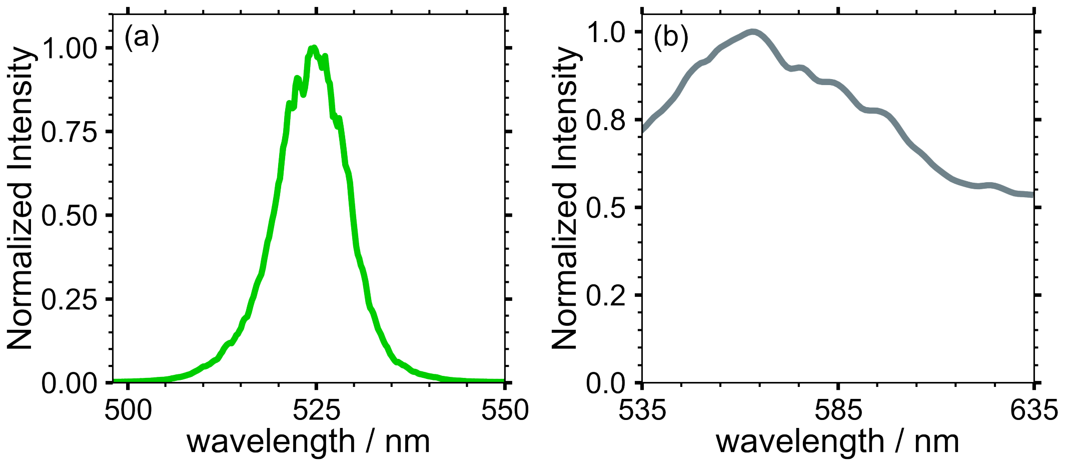

Figure S2. (a) Spectrum of pump pulses used in single-shot transient absorption measurements. (b) Spectrum of broadband probe across the measured wavelength range.
